# Developmental Shift of Inhibitory Transmitter Content at a Central Auditory Synapse

**DOI:** 10.3389/fncel.2017.00211

**Published:** 2017-07-19

**Authors:** Jana Nerlich, Rudolf Rübsamen, Ivan Milenkovic

**Affiliations:** ^1^Department of Physiology, Faculty of Medicine, Carl Ludwig Institute for Physiology, University of Leipzig Leipzig, Germany; ^2^Faculty of Biosciences, Pharmacy and Psychology, University of Leipzig Leipzig, Germany

**Keywords:** Inhibition, GABA, glycine, corelease, development, mIPSC, auditory brainstem

## Abstract

Synaptic inhibition in the CNS is mostly mediated by GABA or glycine. Generally, the use of the two transmitters is spatially segregated, but there are central synapses employing both, which allows for spatial and temporal variability of inhibitory mechanisms. Spherical bushy cells (SBCs) in the mammalian cochlear nucleus receive primary excitatory inputs through auditory nerve fibers arising from the organ of Corti and non-primary inhibition mediated by a dual glycine-GABA transmission. Slow kinetics IPSCs enable activity dependent tonic-like conductance build up, functioning as a gain control by filtering out small or temporally imprecise EPSPs. However, it remained elusive whether GABA and glycine are released as content of the same vesicle or from distinct presynaptic terminals. The developmental profile of quantal release was investigated with whole cell recordings of miniature inhibitory postsynaptic currents (mIPSCs) from P1–P25 SBCs of Mongolian gerbils. GABA is the initial transmitter eliciting slow-rising and -decaying events of relatively small amplitudes, occurring only during early postnatal life. Around and after hearing onset, the inhibitory quanta are predominantly containing glycine that—with maturity—triggers progressively larger and longer mIPSC. In addition, GABA corelease with glycine evokes mIPSCs of particularly large amplitudes consistently occurring across all ages, but with low probability. Together, these results suggest that GABA, as the primary transmitter released from immature inhibitory terminals, initially plays a developmental role. In maturity, GABA is contained in synaptic vesicles only in addition to glycine to increase the inhibitory potency, thereby fulfilling solely a modulatory function.

## Introduction

GABA and glycine are the major inhibitory neurotransmitters shaping neuronal activity in the mammalian CNS. While cortical and midbrain neurons are mainly inhibited by GABA, inhibition in the brainstem and spinal cord is predominantly mediated by glycine (Fritschy et al., 1994; Legendre, 2001; Huang et al., 2007; Lehmann et al., 2012). However, a number of specialized presynaptic terminals in the cerebellum (Dumoulin et al., 2001; Rousseau et al., 2012; Husson et al., 2014), brainstem (Russier et al., 2002; Dufour et al., 2010; Apostolides and Trussell, 2014) and spinal cord (Jonas et al., 1998; O’Brien and Berger, 1999; Keller et al., 2001; Seddik et al., 2007) engages glycine and GABA signaling in terms of synaptic vesicle content, co-release and activation of respective postsynaptic receptors (Triller et al., 1987; Burger et al., 1991; Bohlhalter et al., 1994; Todd et al., 1996; Dumba et al., 1998; Jonas et al., 1998). Such co-transmission allows for an additional variability, enabling target dependent use of transmitters at the same cell (Chéry and de Koninck, 1999; Nerlich et al., 2014b), at different cells (Dugué et al., 2005; Kuo et al., 2009), or fine-tuning of inhibitory strength and its time course (Russier et al., 2002; Awatramani et al., 2005; González-Forero and Alvarez, 2005; Lu et al., 2008; Coleman et al., 2011; Apostolides and Trussell, 2013; Nerlich et al., 2014b). Synaptic inhibition in mature auditory brainstem circuits, playing an important role in encoding acoustic cues used for sound source localization, can be almost exclusively attributed to the action of glycine (Grothe, 2003; Awatramani et al., 2004; Magnusson et al., 2005; Kopp-Scheinpflug et al., 2008; Pecka et al., 2008; Couchman et al., 2010; Friauf et al., 2011; Roberts et al., 2013; Myoga et al., 2014). In the mammalian cochlear nucleus, however, the activity of spherical bushy cells (SBCs) that receive large glutamatergic auditory nerve fiber terminals, is shaped by a dual glycine-GABA transmission (Nerlich et al., 2014a). Here, a synergistic action of both transmitters elevates the EPSP threshold required for AP generation, thereby providing an activity-dependent gain control (Kuenzel et al., 2011, 2015; Xie and Manis, 2013; Nerlich et al., 2014b; Keine et al., 2016).

Still, it remained elusive whether GABA and glycine are released from distinct presynaptic terminals or coreleased from a single terminal as a content of the same vesicle. Here, we used whole cell recordings to investigate the developmental profile of GABA and glycine quantal release onto SBCs of Mongolian gerbils. Spontaneous miniature inhibitory postsynaptic currents (mIPSCs) show a gradual developmental shift from predominantly GABA-containing to predominantly glycine-containing vesicles between P1 and P25. Before hearing onset (P2–8) mIPSC properties are largely determined by GABA, while the inhibitory quanta shortly before and after hearing onset are predominantly containing glycine. In addition, the data reveal a co-release of GABA and glycine occurring in a subset of vesicles across all ages. With increasing age, particularly mIPSCs with large amplitudes are elicited by vesicles containing GABA in addition to glycine.

## Materials and Methods

The experimental procedures were approved by the Saxonian district Government Leipzig (T 67/13, T 34/16) and conducted in agreement with regulations applying to the University of Leipzig according to the European Communities Council Directive (2010/63/EU). Mongolian gerbils (*Meriones unguiculatus*) were bred at the animal facility of the Faculty of Biosciences, Pharmacy and Psychology, University of Leipzig. Animals had *ad libitum* access to food and water and grew under a 12/12 h day/night cycle. Authors have conducted all available measures to minimize animals’ pain and suffering.

### Slice Preparation

Coronal slices (200 μm), containing the rostral AVCN, were cut from P1–P30 gerbils of either sex. The brainstem was sliced with a vibratome (Microm HM 650), in low-calcium artificial cerebrospinal fluid (ACSF) solution containing (in mM): 125 NaCl, 2.5 KCl, 0.1 CaCl_2_, 3 MgCl_2_, 1.25 NaH_2_PO_4_, 25 NaHCO_3_, 25 glucose, 2 sodium pyruvate, 3 myo-inositol, 0.5 ascorbic acid, continuously bubbled with 5% CO_2_ and 95% O_2_, pH 7.4. Slicing solution contained lower Ca^2+^ and higher Mg^2+^ concentration than the standard ACSF in order to avoid Ca^2+^-dependent signaling and activation of NMDAR. Incubation of slices was done in the standard recording ACSF containing 125 NaCl, 2.5 KCl, 2 CaCl_2_, 1 MgCl_2_, 1.25 NaH_2_PO_4_, 25 NaHCO_3_, 10 glucose, 2 sodium pyruvate, 3 myo-inositol, 0.5 ascorbic acid, continuously bubbled with 5% CO_2_ and 95% O_2_, pH 7.4, for 30 min at 37°C. Thereafter, slices were stored at room temperature until recordings performed at nearly physiological temperature (33.5 ± 0.3°C).

### Electrophysiological Recordings

Whole-cell patch clamp recordings were performed on SBCs in the rostral pole of the AVCN. Morphological verification of recorded neurons was done by intracellular labeling with ATTO 488 (ATTO-TEC GmbH, Cat.No. AD 488-21) and visualization with a CCD camera (IMAGO Typ VGA; TILL Photonics). Neurons from P1-P4 animals could not be reliably identified as SBCs because of their immature morphology. Cells recorded in P7-P30 animals exhibit the typical morphology of large SBCs with oval cell soma and a developing dendritic tree containing one or few primary dendrites terminating in short and bushy dendritic arbors. The pipettes had resistances of 3.3 ± 0.7 MΩ (mean ± SD) when filled with (mM): 107 CsCl, 18 TEA-Cl, 1 MgCl_2_, 20 HEPES, 5 EGTA, 4.5 QX-314-Cl, 5 phosphocreatine, 2 ATP disodium salt, 0.3 GTP disodium salt, and 50 μM ATTO 488 (pH 7.3 with CsOH). Voltage clamp measurements were done from V_hold_ = −70 mV. The resulting spontaneous miniature IPSCs had larger amplitudes than events recorded with [Cl^−^]_pip_ = 28 mM (estimated [Cl^−^]_i_ around P3–5, Witte et al., 2014). While the analyses of such larger mIPSC enables reliable conclusions, different [Cl^−^]_pip_ had no effect on mIPSC decay time constants (mean mIPSC τ decay ± SD at P2–4 [Cl^−^]_pip_ = 131 mM: 17.5 ± 5.6 ms, *n* = 14, [Cl^−^]_pip_ = 28 mM: 14.8 ± 3.5 ms, *n* = 6; P7–8 [Cl^−^]_pip_ = 131 mM: 17.2 ± 4.8 ms; *n* = 13, [Cl^−^]_pip_ = 28 mM: 15.2 ± 5.3 ms; *n* = 5; P23–25 [Cl^−^]_pip_ = 131 mM: 17.3 ± 4.2 ms; *n* = 13, [Cl^−^]_pip_ = 28 mM: 17.2 ± 1.4 ms; *n* = 8; effect of [Cl^−^]_pip_ *p* = 0.7 and age *p* = 0.2, interaction between [Cl^−^]_pip_ and age *p* = 0.6, two way ANOVA). In addition, the decay of mIPSCs recorded in P23–25 SBCs with [Cl^−^]_pip_ = 5 mM (estimated [Cl^−^]_i_ after hearing onset, Milenković and Rübsamen, 2011) exhibited comparable τ decay of 19.7 ± 3.0 ms (*n* = 3).

IPSCs were recorded with [Cl^−^]_pip_ = 28 mM (125 CsMeSO_3_, 18 TEA-Cl, 3 MgCl_2_, 10 HEPES, 0.1 EGTA, 4.5 QX-314-Cl, 5 phosphocreatine, 2 ATP disodium salt, 0.3 GTP disodium salt, pH 7.3 with CsOH) to enable comparison with previously published data (Nerlich et al., 2014a,b). Briefly, electrical stimulation of afferent fibers was conducted through a bipolar theta glass electrode (Science Products, tip Ø 5 μm) filled with bath solution and placed randomly at distances of 30–60 μm from the cell. Pulse stimuli (100 μs, 15–90 V) were generated by a stimulator (Master 8) and delivered via a stimulus isolation unit (AMPI Iso-flex) to evoke either single events or train-responses at 100 Hz.

Glycine receptor (GlyR) and GABA_A_ receptor (GABA_A_R) mediated mIPSCs were recorded under pharmacological inhibition of glutamate- (50 μM AP-5, 10 μM NBQX) and GABA_B_-receptors (3 μM CGP 55845), and voltage-gated sodium channels (0.5 μM TTX). A liquid junction potential of 2 mV was calculated using Clampex (Molecular Devices) and voltages were accordingly corrected offline (Neher, 1992).

Glycine and GABA (500 μM) were prepared in standard recording ACSF and pressure applied over the soma of recorded neuron using a Picospritzer (General Valve Corp.). As in all other experiments, ACSF was supplemented with the GABA_B_ antagonist CGP 55845. The constant stimulation conditions were assured by controlling the pipette diameter, application pressure and duration, and distance from the cell (3 μm, 5 psi, 5 ms, 10 μm, respectively). The perfusion was turned off just prior to each puff application to avoid unequal dilution of the agonist. Significance of the responses was determined by employing the *z*-test, i.e., level of acceptance was set at *z* < −3.3 (I_m_), which corresponds to *p* < 0.001 (*z* = (A-BL)/SD_BL_, with A being the maximal amplitude of the response, BL the mean of the baseline (2 s prior to stimulation), SD_BL_ the standard deviation of the baseline). Pressure-ejection of ACSF under the same condition evoked no response (mean ACSF response = −0.8 ± 2.7 pA, *z* = −0.1 ± 0.2, *p* > 0.26, *n* = 9), while glycine and GABA evoked significant membrane currents in the same neurons.

### Data Acquisition and Analysis

The recordings were acquired using a Multiclamp 700B amplifier (Molecular Devices). The average series resistance was 9.3 ± 2.9 MΩ (mean ± SD, *n* = 113 SBCs), which was compensated by 70%–80% to a remaining Rs of 2.7 ± 0.8 MΩ. During experiments the series resistance changed on average by 3.6%. Cells with series resistance changes >15% were excluded from analysis. Recorded signals were digitized at 50 kHz and filtered with a 5 kHz Bessel low-pass filter. Data were examined with pClamp10 software (Molecular Devices) and MATLAB based scripts (version 2015a, The MathWorks). mIPSCs were detected using a template search method (Clampfit 10.2, Molecular Devices) when crossing a detection threshold of four times the standard deviation of the baseline. False positive noise-triggered fluctuations and signals with a non-monotonic rising phase and/or additional events within the decay phase were rejected on visual inspection. Peak amplitudes, 10%–90% rise times, and τ decay of peak-aligned mIPSCs were further analyzed using custom-written MATLAB routines. mIPSC decay phase was fitted with mono- or bi-exponential function based on an increase of adjusted *R*^2^ values. The weighted τ decay was calculated as τ_wd_ = (A_fast_ × τ_fast_ + A_slow_ × τ_slow_)/(A_fast_ + A_slow_), where A_fast_ and A_slow_ are amplitudes at *t* = 0 and τ_fast_ and τ_slow_ are the fast and slow time constants, respectively. In cases of mono-exponential fits, one exponential component was set to 0. For the present data, an increase of adjusted *R*^2^ using the bi-exponential fit indicates that the better fit is justified by the increased number of coefficients in the model, while a decrease in adjusted *R*^2^ indicates that the additionally introduced coefficients do not provide a better fit to the data than would be expected by chance (Anderson-Sprecher, 1994).

### Immunohistochemistry

Gerbils were euthanized with CO_2_ inhalation and the tissue was fixed through transcardial perfusion with 4% paraformaldehyde in PBS. The brains were taken from two maturity stages (P25 and P60) and prepared in parallel to assure the same experimental conditions. The morphological development of inhibitory terminals on SBCs, including their transmitter content, seems to be completed by P21 (Luján et al., 2008). Nevertheless, P60 gerbils were used here to compare the results between adult and sub-adult P25 animals. Staining conducted in pre-hearing gerbils (P2–8) could not be reliably quantified, presumably due to poor fixation of the immature tissue.

Following transcardial perfusion with 4% PFA, the brain was removed from the skull and post-fixed overnight at 4°C. Coronal brainstem sections (30 μm) containing the rostral AVCN were cut using a vibratome. After blocking of nonspecific binding sites with 5% normal donkey serum in PBS/0.2% Triton X-100 (60 min at 37°C), free-floating sections were incubated overnight at 4°C with a guinea pig anti-GlyT2 antibody (1:5000, Millipore, Cat. No. AB1773), a mouse anti-GAD65/67 antibody (1:1000, Biotrend, Cat. No. MSA-225E), and a rabbit anti-MAP2 (1:250, Synaptic Systems, Cat. No. 188 003). The primary antibodies against GlyT2 and GAD65/67 correspond to those published previously (Dufour et al., 2010; Husson et al., 2014). Alternatively, a rabbit anti-GAD65/67 antibody (1:1000, Sigma-Aldrich, Cat. No. G5163, Dixon and Harper, 2001; Brückner et al., 2008), and a mouse anti-gephyrin antibody (1:500, Synaptic System, Cat. No. 147021, Dufour et al., 2010) were used for triple staining along with guinea pig anti-GlyT2. After wash with PBS/0.2%Triton X-100, the slices were incubated with secondary donkey anti-guinea pig Alexa fluor 488 (1:400), donkey anti-mouse Cy3 (1:800) and donkey anti-rabbit Alexa fluor 647 (1:800; all from Jackson Immunoresearch Lab. Dianova, Hamburg, Germany) for 2.5 h at RT. Following rinsing with PBS and dH_2_O, the sections were coverslipped with aqua-Polymount. The staining with well characterized anti-MAP 2 antibody (Michalski et al., 2013; Härtig et al., 2016) was conducted to visualize SBCs, thereby highlighting the presynaptic localization of GlyT2 and GAD65/67. MAP 2 staining was color-coded in blue to enhance the contrast to the red GAD65/67 labeling. Each staining procedure was controlled by the omission of primary antibodies and the subsequent identical processing of a few sections. Due to the lack of fluorescence in control experiments, the appropriate focus-settings were confirmed through additional image acquisition with transmission light. Sections were examined with a confocal laser-scanning microscope (TCS PS5, Leica).

### Colocalization Analysis

The colocalization of presynaptic markers for glycinergic and GABAergic terminals was analyzed from triple immunostainings for GlyT2-GAD65/67-MAP2. For this, background subtracted images were imported into ImageJ where the regions of interest (ROI) were marked around MAP2-positive SBC’s somata using ImageJ’s region of interest manager. Then, a square area containing ROI with the cell soma was cropped from a single image of a confocal stack for further analysis with ImageJ’s colocalization threshold plug in. The intensity threshold for each channel was set by the algorithm using the Costes automatic thresholding method (zero-zero pixels were excluded in threshold calculation). Pixels below threshold were ignored for quantification. Colocalization of GAD65/67 and GlyT2 was expressed as the colocalization coefficients. They represent the proportion of colocalized pixels in channel 1 (GAD65/67) or channel 2 (GlyT2) of the composite image, relative to the total number of pixels above the threshold in that channel. To consider a pixel as colocalized, the intensities in both channels had to exceed their respective thresholds. The number of colocalized pixels compared to the total number of pixels above threshold in each channel was used to evaluate the amount of colocalization of GAD65/67 and GlyT2 immunoreactivity. This is considered as a quantitative measure of presynaptic terminals coreleasing GABA and glycine.

### Statistics

Data sets were tested for Gaussian distribution prior to comparison with paired two-sample *t*-test or analysis of variance (ANOVA). Within-subject comparisons were performed by two-way repeated-measures (RM) ANOVA after testing for sphericity using Mauchly test, and applying Greenhouse-Geissner correction where appropriate to increase the accuracy of significance values. The *p* values of pairwise comparisons were adjusted by the Dunn-Šidak procedure. In case of a non-Gaussian distribution, the non-parametric Kruskal-Wallis ANOVA was applied. Cumulative distribution of mIPSC amplitudes and kinetics were compared before and after drug application and between age groups using the non-parametric two-sample Kolmogorov-Smirnov test (K-S test). Statistical analyses were carried out in MATLAB using the statistics toolbox (version 2016a, The MathWorks) and in Sigma Plot (Sigma Plot 11, Systat Software). Values are reported as mean ± SEM or median (first quartile, third quartile) based on the data distribution.

## Results

### Developmental Increase in Spontaneous Vesicle Release and Quantal Size

**Figure 1:**
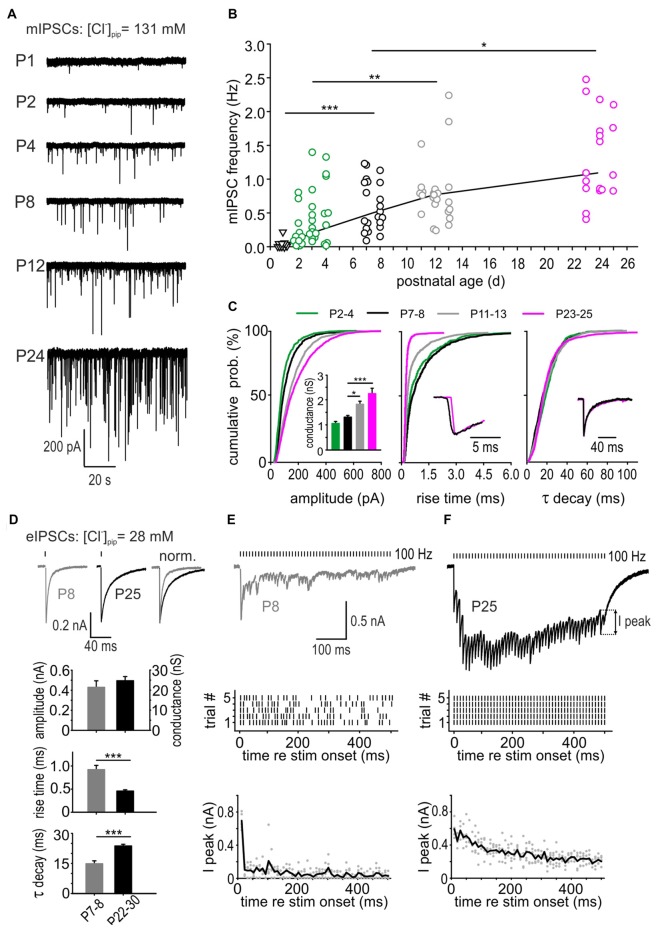
Developmental changes in vesicular release. **(A)** Example traces of miniature inhibitory postsynaptic currents (mIPSCs) from different postnatal ages recorded in the presence of 0.5 μM tetrodotoxin (TTX). **(B)** Developmental increase of mIPSC frequency in spherical bushy cells (SBCs). Each symbol represents the value of a single cell. The solid black line connects the median mIPSC frequencies for age groups P1 (triangles), P2–4 (green circles), P7–8 (black circles), P11–13 (gray circles) and P23–25 (magenta circles; effect of age *p* < 0.001, analysis of variance (ANOVA), *post hoc* pairwise comparisons: *p < 0.05, **p < 0.01, ***p < 0.001). Miniature IPSCs occurring at low frequency could be detected in 50% of the cells at P1. **(C)** Mean cumulative distribution functions (CDFs) of mIPSC amplitudes (left), 10%–90% rise times (middle) and decay time constants (right) obtained from different age groups. The mIPSC amplitudes increased and the rise times accelerated up to P23–25 (all pairwise comparisons: *p* < 0.001, K-S tests). The decay time constant showed no developmental change. Inset (left) shows mean ± SEM conductances at different age groups calculated from mIPSC amplitudes obtained in each cell; *p < 0.05, ***p < 0.001, ANOVA). Insets (middle, right) show median mIPSCs at P7 (black) and P24 (magenta). Traces were normalized to the peak to highlight the differences in mIPSC kinetics. **(D)** Representative IPSC traces evoked by single stimulation of inhibitory inputs in a P8 (left) and a P25 SBC (middle). Traces on the right were peak-normalized to highlight the difference in decay. Mean eIPSC amplitudes and conductances were similar for P7–8 and P22–30 cells (*n* = 16, *n* = 43, respectively). Note the shorter rise time and longer decay time in maturity (*p* < 0.001, *t*-test). **(E,F)** Top: IPSCs evoked by a repetitive stimulation (100 Hz, 50 pulses) at P8 and P25. Middle: raster plots showing the temporal precision of the IPSCs evoked during five repetitions. Each line represents the time point of a single peak response. Note the failures in a P8 SBC, particularly occurring late in the train. At P25 each stimulus reliably evoked a response. Bottom: developmental decrease of peak-amplitude depression. I-peak amplitudes were calculated as foot-to-peak for each IPSC throughout the train and plotted for representative cells from P8 and P25 (gray dots). Black lines show average for amplitudes elicited during five repetitions. Note the pronounced depression after few initial events at P8, which is much less pronounced at P25.

Spontaneous vesicle release was measured under superfusion of 0.5 μM TTX to block the action potential-triggered release. To reveal the developmental changes of mIPSC properties, data were grouped according to landmarks in the development of inhibition in the AVCN: P2–4 (depolarizing action of GABA and glycine in SBCs, Milenković et al., 2007; Witte et al., 2014), P7–8 (shift to hyperpolarizing effect, Milenković et al., 2007), P11–13 (period of onset of acoustically evoked signal processing [i.e., hearing onset], Woolf and Ryan, 1985), P22–25 (adult like morphology of inhibitory inputs, Luján et al., 2008). Example recordings in Figure 1A show a continuous developmental increase in mIPSC frequency and amplitude. Miniature IPSCs occurring at low frequency were detectable at P1, but only in a subset of neurons. The frequency of mIPSC increased 6-fold between P2–4 and P23–25 (Figure 1B, mIPSC frequency in Hz P1: 0.01 [0; 0.03], *n* = 10; P2–4: 0.18 [0.05; 0.47], *n* = 37; P7–8: 0.48 [0.29; 0.95], *n* = 22; P11–13: 0.75 [0.54; 0.84], *n* = 25, P23–25: 1.08 [0.85; 1.85], *n* = 18; effect of age: *p* < 0.001, comparison across age groups: P1 vs. P7–8: *p* < 0.001, P2–4 vs. P11–13: *p* < 0.01, P7–8 vs. P23–25 *p* < 0.05, ANOVA). Mean cumulative distribution functions (CDFs) representing diverse mIPSC properties were generated by analyzing equal numbers of randomly picked mIPSCs from each cell to ensure an equivalent contribution to the population data. The mean CDF of mIPSC amplitudes was significantly shifted toward higher values with increasing maturity stages (Figure 1C left; P2–4: 560 events, *n* = 14; P7–8: 650 events, *n* = 13; P11–13: 1200 events, *n* = 15; P23–25: 1040 events, *n* = 13; all comparisons: *p* < 0.001, K-S tests). Within a single cell, the values for mIPSC amplitudes, rise times and τ decay showed a non-Gaussian distribution. Therefore, the respective parameters were calculated as a median value per cell. However, as the median values for all cells within one age group followed a Gaussian distribution, the population data are presented as mean ± SEM. Events recorded at P23–25 exhibited on average two-fold larger amplitudes than events at P2–4 (mIPSC amplitude in pA P2–4: 74.0 ± 5.5, P7–8: 91.6 ± 4.4, P11–13: 127.6 ± 8.5, P23–25: 157.1 ± 15.0; effect of age *p* < 0.001, P7–8 vs. P11–13, *p* < 0.05, P7–8 vs. P23–25: *p* < 0.001, ANOVA). The parameters describing kinetics of mIPSCs, such as 10%–90% rise time and τ decay, were differently affected by age. The mIPSC rise time shortened by half during the first 4 postnatal weeks (rise time in ms P2–4: 0.54 ± 0.08, P7–8: 0.70 ± 0.11, P11–13: 0.37 ± 0.04, P23–25: 0.23 ± 0.01; effect of age *p* < 0.001, P7–8 vs. P11–13, *p* < 0.01, P7–8 vs. P23–25: *p* < 0.001, ANOVA; Figure 1C, all CDF comparisons: *p* < 0.001, K-S test). Contrary to the rise time, the decay time showed no developmental change (τ decay in ms P2–4: 17.5 ± 1.5, P7–8: 17.2 ± 1.3, P11–13: 17.1 ± 1.4, P23–25: 17.3 ± 1.2; effect of age *p* = 0.99, ANOVA). Together, these data show a developmental increase in spontaneous vesicular release, the increased transmitter content of inhibitory vesicles and the shorter rise time of mIPSCs.

To relate the changes in unitary quantal properties to synaptic events evoked by multiquantal release through presynaptic action potentials, electrical stimulation was used to record pharmacologically isolated eIPSCs in P7–8 and P22–30 SBCs (Figure 1D). The amplitudes of single event eIPSCs were similar between the two age groups (mean eIPSC amplitude in pA P7–8 = −429.5 ± 64.4, *n* = 16; P22–30 = −494.8 ± 41.2, *n* = 43, *p* = 0.46, *t*-test). However, the kinetics of eIPSCs underwent similar developmental changes as mIPSCs, i.e., shortening of the rise time and prolonging of τ decay (rise time in ms P7–8 = 0.92 ± 0.09, *n* = 16; P22–30 = 0.46 ± 0.03, *n* = 43, *p* < 0.001; τ decay in ms P7–8 = 14.9 ± 1.4, *n* = 16; P22–30 = 23.7 ± 0.8, *n* = 43, *p* < 0.001, *t*-test). Next, it was examined how well the inhibitory synapses at two different maturity stages follow 100 Hz synaptic activity during 50-pulses. Representative traces from a P8 (Figure 1E) and a P25 SBC (Figure 1F) demonstrate conspicuously better synaptic efficacy in maturity. At P8, eIPSCs are either occurring with increased latency (time between stimulation artifact and eIPSC peak) and prominent jitter (SD of the latency), or the synapses fail to initiate a detectable eIPSCs (Figure 1E, top and middle, average latency ± jitter in ms for the first five IPSCs and five repetitions P8 = 2.66 ± 1.30; P25 = 1.49 ± 0.14, *p* < 0.001; last five IPSCs P8 = 3.37 ± 1.31; P25 = 1.61 ± 0.17, *p* < 0.001, *t*-test). In addition, foot-to-peak IPSC amplitudes are strongly depressing after the first few events in P8 SBCs (Figure 1E, bottom). Contrary to this, synaptic activity at P25 elicits temporally precise eIPSCs occurring with high reliability and with less depression (Figure 1F). Comparison of failure rates for the first and the last five events in a 50-pulse train shows significant differences between P8 and P25 SBCs (average failure rate first five IPSCs P8 = 14 ± 5%, P25 = 0%, *p* = 0.018; last five IPSCs P8 = 80 ± 7%, P25 = 9 ± 3%, *p* < 0.001; first five vs. last five P8 *p* < 0.001, P25 *p* = 0.09; two way ANOVA).

Based on the data from mIPSCs and eIPSCs recordings, it can be concluded that increased transmitter content, number of vesicles, and synchrony of release contribute to maturation of synaptic machinery required to maintain ongoing synaptic activity.

### Developmental Change of GABA and Glycine Contributions to the Inhibitory Vesicle Content

**Figure 2:**
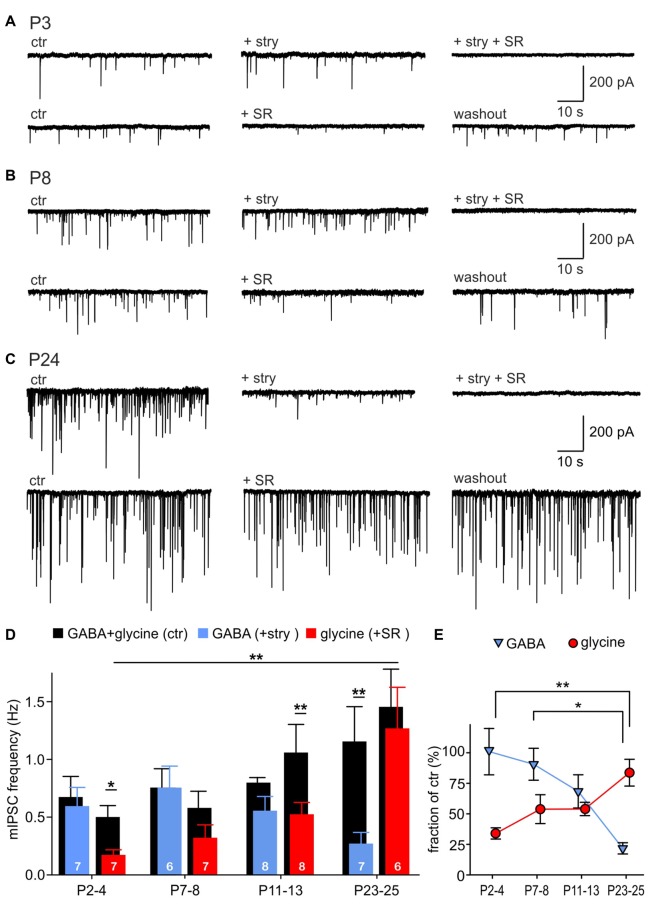
Developmental shift of inhibitory vesicle content. **(A–C)** Example recordings of mIPSCs at P3 **(A)**, P8 **(B)** and P24 **(C)**. mIPSCs were differently affected by 0.5 μM strychnine or 15 μM SR95531 depending on the maturity. Combination of both antagonists completely blocked mIPSCs. **(D)** Mean frequencies of isolated glycinergic and GABAergic mIPSCs and respective controls at different ages (mean ± SEM; *p < 0.05, **p < 0.01, two-way repeated-measures (RM) ANOVA, cell numbers are given at the bottom of the bars). **(E)** Relative GABAergic and glycinergic contributions to the total mIPSC frequency at different ages (mean ± SEM; *p < 0.05, **p < 0.01, ANOVA).

To examine the relative contribution of GABA and glycine to mIPSCs across development, the events evoked by GABA were pharmacologically isolated by superfusion of 0.5 μM strychnine, or the events elicited by glycine were isolated with 15 μM SR95331 (Figures 2A–C). Example recordings shown in Figures 2A–C reveal a gradual developmental shift from predominantly GABA-containing to predominantly glycine-containing vesicles. This is demonstrated by weak inhibitory effects of strychnine at P3 (Figure 2A) and of SR95331 at P24 (Figure 2C). Notably, the average frequency of isolated GABAergic mIPSC remained stable throughout development (Figure 2D; P2–4: 0.60 ± 0.16 Hz, *n* = 7; P7–8: 0.32 ± 0.11 Hz, *n* = 6; P11–13: 0.53 ± 0.10 Hz, *n* = 8; P23–25: 1.27 ± 0.36 Hz, *n* = 7; all pairwise comparisons: *p* > 0.05, two way RM ANOVA). However, because of the increasing mIPSC frequency under control condition, the relative GABAergic fraction (after pharmacological inhibition of GlyR) decreased significantly from on average 100.9 ± 19.0% at P2–4 to 21.7 ± 4.6% at P23–25 (Figure 2E; effect of age: *p* = 0.004, P2–4 vs. P23–25: *p* < 0.01, P7–8 vs. P23–25: *p* < 0.05, ANOVA). Hence, the average GABAergic mIPSC frequency was similar to the controls in the first postnatal week, but substantially smaller than controls in the fourth postnatal week (Figure 2D, P23–25: ctr vs. GABA, *p* = 0.007, two way RM ANOVA). On the other hand, the incidence of glycinergic events increased remarkably with age (Figure 2D, P2–4: 0.17 ± 0.04 Hz, *n* = 7; P7–8: 0.32 ± 0.11 Hz, *n* = 7; P11–13: 0.53 ± 0.10 Hz, *n* = 8; P23–25: 1.27 ± 0.36 Hz, *n* = 6; P2–4 vs. P23–25: *p* < 0.01, two way RM ANOVA). The contribution of glycine to the total mIPSC frequency increased from 33.9 ± 4.6% at P2–4 to 83.7 ± 11.0% at P23–25, indicating that glycine mediates the major inhibitory effect in maturity (Figure 2E, effect of age: *p* = 0.003, P2–4 vs. P23–25: *p* < 0.01, ANOVA). During the early postnatal period (P2–4 and P7–8), summation of the isolated GABAergic and glycinergic fractions results in a supra-additive mIPSC frequency (135 and 144%), i.e., larger than the frequency measured under control condition. As the mIPSCs frequency did not change under strychnine between P2 and P8, it can be concluded that all mIPSCs contain a GABAergic component. Together, these data suggest that early after birth all vesicles are likely to contain GABA, and a small subset of vesicles in addition contains glycine. Around hearing onset, glycine and GABA were assessed in about 54% and 68% of vesicles, respectively. Accordingly, it is conceivable that GABA and glycine are released from separate vesicles, but a corelease of both in a subset of vesicles cannot be ruled out. At P23–25 the fractions of isolated GABAergic (21.7 ± 4.6%) and glycinergic events (83.7 ± 11.0%) add up to ~100%, which represents the total mIPSC frequency. This may imply a release of GABA and glycine from different vesicles. However, as the pharmacological inhibition of GABA_A_R evoked no frequency change, it also can be inferred that vesicles containing only GABA are not likely to occur (Figure 2D, P23–25 ctr vs. SR: *p* = 0.2, two way RM ANOVA). Hence, a corelease of GABA and glycine can be assumed in a subset of vesicles. Taken together, these data suggest that GABA release does hardly change during development, but, due to an increment of glycine release, GABA-containing synaptic vesicles have a low probability of occurrence in maturity.

**Figure 3:**
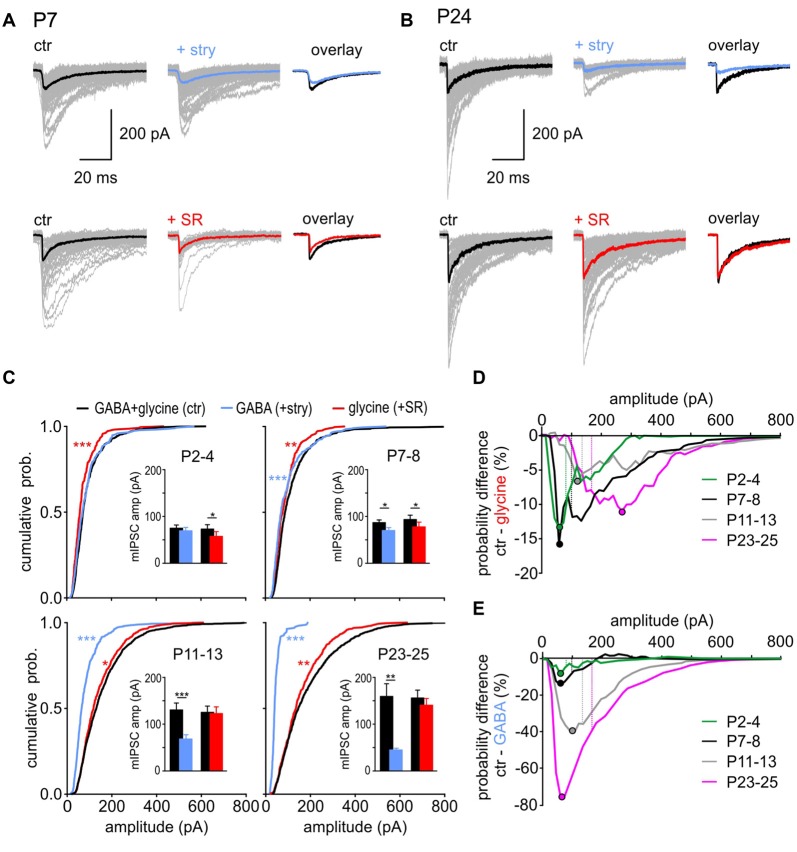
GABA and glycine contribution to mIPSC amplitudes. **(A,B)** Superimposed individual mIPSCs (gray), with respective median for control (black), GABAergic (blue) and glycinergic (red) events from representative SBCs at P7 **(A)** and P24 **(B)**. Right: overlay of the median mIPSCs before and after the respective drug application. **(C)** Left: mean CDFs of control, GABAergic, and glycinergic mIPSC amplitudes at different postnatal ages (ctr vs. transmitter: *p < 0.05, **p < 0.01, ***p < 0.001, K-S test). Insets show summary data for median amplitudes before and after block of glycine receptor (GlyR) (+stry) or GABA_A_R (+SR) (bars represent the mean amplitude of the respective age group ± SEM calculated from the median amplitudes per cell; transmitter vs. respective control: *p < 0.05, **p < 0.01, ***p < 0.001, paired *t* test). **(D,E)** Difference curves obtained by subtraction of glycinergic CDF **(D)** or GABAergic CDF **(E)** from control CDFs. The curves show the probability difference for events evoked by each transmitter compared to control events. Filled circles show the mIPSC amplitude at which the maximal difference was observed between the isolated glycine **(D)** or GABA **(E)** events and the respective control. For better comparison, dashed lines represent the mean mIPSC amplitude of control cells for each age group.

In addition to the age-dependent change in incidence of GABA-containing and glycine-containing events, the respective transmitters also contributed differently to the total of mIPSC amplitudes during development. In a single cell, they don’t reveal a Gaussian distribution, due to the small number of prominently larger events. Figures 3A,B show peak-aligned mIPSCs at P7 and P24 with color-coded traces representing the median for each condition. At P7, the median amplitude of pharmacologically isolated GABAergic or glycinergic events is smaller than the median of control mIPSCs (Figure 3A). These data suggest that the events with larger amplitudes occurring under control condition are evoked by a corelease of GABA and glycine from a single vesicle. At P24, glycine mostly determines the mIPSC amplitude, as shown by the strong effect of strychnine disclosing isolated GABAergic events with conspicuously small amplitudes (Figure 3B *top*). However, as the median amplitude under SR95531 did not differ from control, the GABAergic component may be only contributing to fewer events with larger amplitudes (Figure 3B *bottom*).

To closely examine how GABA and glycine contribute to mIPSC amplitudes at different developmental stages, the data were analyzed as cumulative distribution plots (Figure 3C). At P2–4, mIPSCs amplitudes of isolated GABAergic events were similar to control (Figure 3C *top left*, CDF comparison: ctr (*n* = 14) vs. GABA (*n* = 7): *p* = 0.3, K-S test; inset: mean values ctr_GABA_ = 74.9 ± 6.8 pA, GABA = 69.3 ± 7.3 pA, *p* = 0.4, *n* = 7, paired *t* test). This indicates that control events are largely determined by GABA across the whole range of amplitudes. After blocking the GABA_A_R, the remaining glycine-mediated events were shifted towards smaller values (CDF comparison: ctr (*n* = 14) vs. glycine (*n* = 7): *p* < 0.001, K-S test; inset: mean values ctr_glycine_ = 73.0 ± 9.3 pA, glycine = 57.3 ± 10.2 pA, *p* = 0.03, *n* = 7, paired *t* test). The following can be concluded from these findings: if glycine is eliciting a larger-amplitude mIPSC, it is likely to happen through a corelease with GABA. At P7–8, pharmacological isolation of GABA-containing and glycine-containing events revealed that mIPSC amplitudes evoked by each of the two transmitters alone are significantly smaller than control events (Figure 3C *top right*, CDF comparisons: ctr (*n* = 13) vs. GABA (*n* = 7): *p* < 0.001; ctr (*n* = 13) vs. glycine (*n* = 6): *p* < 0.01, K-S test; inset: mean values ctr_GABA_ = 87.7 ± 6.3 pA, GABA = 71.0 ± 5.9 pA, *p* = 0.05, *n* = 7; ctr_glycine_ = 94.0 ± 7.4 pA, glycine = 77.2 ± 10.3 pA, *p* = 0.02, *n* = 6, paired* t* test). Considering that at P7–8 all vesicles are likely to contain GABA, and about 50% of vesicles in addition glycine (Figure 2E), mIPSCs of larger amplitudes occurring under control condition are probably elicited through corelease. With maturation, the contribution of glycine became more prominent and both at P11–13 and at P23–25, the mean amplitudes of glycinergic mIPSCs were similar to control (Figure 3C *bottom*, P11–13: ctr_glycine_ = 125.5 ± 11.7 pA, glycine = 122.9 ± 13.7 pA, *p* = 0.55, *n* = 8; P23–25: ctr_glycine_ = 155.6 ± 17.1, glycine = 140.6 ± 14.4, *p* = 0.12, *n* = 8, paired *t* test). However, analyses of CDF plots revealed that isolated glycinergic events are lacking the mIPSCs with larger amplitudes present under control condition (P11–13: ctr (*n* = 15) vs. glycine (*n* = 8): *p* < 0.05; P23–25: ctr (*n* = 13) vs. glycine (*n* = 8), *p* = 0.006, K-S test). Accordingly, the glycine-containing vesicles evoking mIPSCs of amplitudes >100 pA are most likely containing GABA as well. This is further supported by the experiments showing that isolated GABA-evoked mIPSCs have on average 2-fold and 3.5-fold smaller amplitudes than control events at P11–13 and P23–25, respectively (CDF comparison: P11–13: ctr (*n* = 15) vs. GABA (*n* = 7): *p* < 0.001; P23–25: ctr (*n* = 13) vs. GABA (*n* = 6), *p* < 0.001, K-S test; mean values P11–13: ctr_GABA_ = 129.9 ± 13.3 pA, GABA = 67.6 ± 8.2 pA, *p* < 0.001, *n* = 8; P23–25: ctr_GABA_ = 158.9 ± 27.6 pA, GABA = 44.61 ± 3.3 pA, *p* < 0.01, *n* = 6, paired *t* test). The data in Figure 3C point out that at P2–4 the control mIPSCs are generally triggered by GABA, then at P7–8 glycine boosts GABAergic mIPSCs, while at P11–13 and P23–25 mIPSCs are predominantly evoked by glycine with an occasional, additional contribution of GABA resulting in particularly large events.

To provide a comprehensive understanding of how the mIPSC size correlates with contribution of GABA and glycine, CDF for pharmacologically isolated events was subtracted from control CDF. The CDF obtained under pharmacological inhibition of GABA_A_R (+SR) represents the cumulative amplitude distribution of isolated glycinergic events. Subtraction of glycinergic from control CDF reveals the difference in probability of occurrence for the whole range of amplitudes (Figures 3D,E). Each dot symbol indicates the mIPSC amplitude at which the maximal probability difference was observed between control and glycinergic events. It can be inferred that isolated glycinergic events of certain amplitudes have lower probability of occurrence than control events, because they are lacking a pharmacologically blocked GABAergic component. The contribution of GABA to the total mIPSC amplitude remained largely constant during development, indicated by a 7%–16% difference in control-glycinergic probability curves (Figure 3D). However, with maturity GABA is contained in vesicles that evoke larger control events. This is demonstrated by a developmental shift in maximal CDF difference towards larger amplitudes (maximal difference at P2–4 at the amplitude of 60 pA; at P23–25 at the amplitude of 270 pA). As the amplitudes of pharmacologically isolated GABAergic events are in the range of 17–187 pA (mean ± SD = 44.6 ± 8.1 pA), the possibility that a sole release of GABA accounts for the difference between control and glycine CDFs for amplitudes >200 pA can be ruled out. Therefore, if GABA is contributing to mIPSCs of large amplitudes, it is likely the result of a corelease with glycine. This notion is further supported by the data showing no mIPSC frequency change under inhibition of GABA_A_R at P23–25 (Figure 2D). Moreover, a fairly consistent probability difference between control and glycine-evoked amplitudes suggests that, across all ages, a subset of glycine-containing vesicles also includes GABA. Contrary to GABA, glycine increasingly contributes to mIPSC amplitudes with postnatal age (Figure 3E). This is evidenced by a progressively larger probability difference between control and GABAergic CDFs during postnatal development (9 vs. 76% for P2–4 and P23–25, respectively). The maximal difference for each age group falls within the mIPSC amplitudes smaller than the mean control amplitude for the respective age group (Figure 3E, maximal deflection depicted by dots vs. mean mIPSC amplitude for age-group depicted by dashed lines). This shows that control events with amplitudes <100 pA are less likely to contain GABA with ongoing maturation. In summary, the contribution of GABA and glycine to mIPSC amplitude shifts during development from predominantly GABA- to predominantly glycine-mediated. From P7–8 on, particularly large events are evoked by vesicles containing both GABA and glycine. This can hardly be inferred from the mean population data, because of the rare occurrence of larger events, but it becomes evident from analysis of CDF plots.

### mIPSCs Are Differently Shaped by GABA_A_- and Glycine-Receptor Kinetics during Development

**Figure 4:**
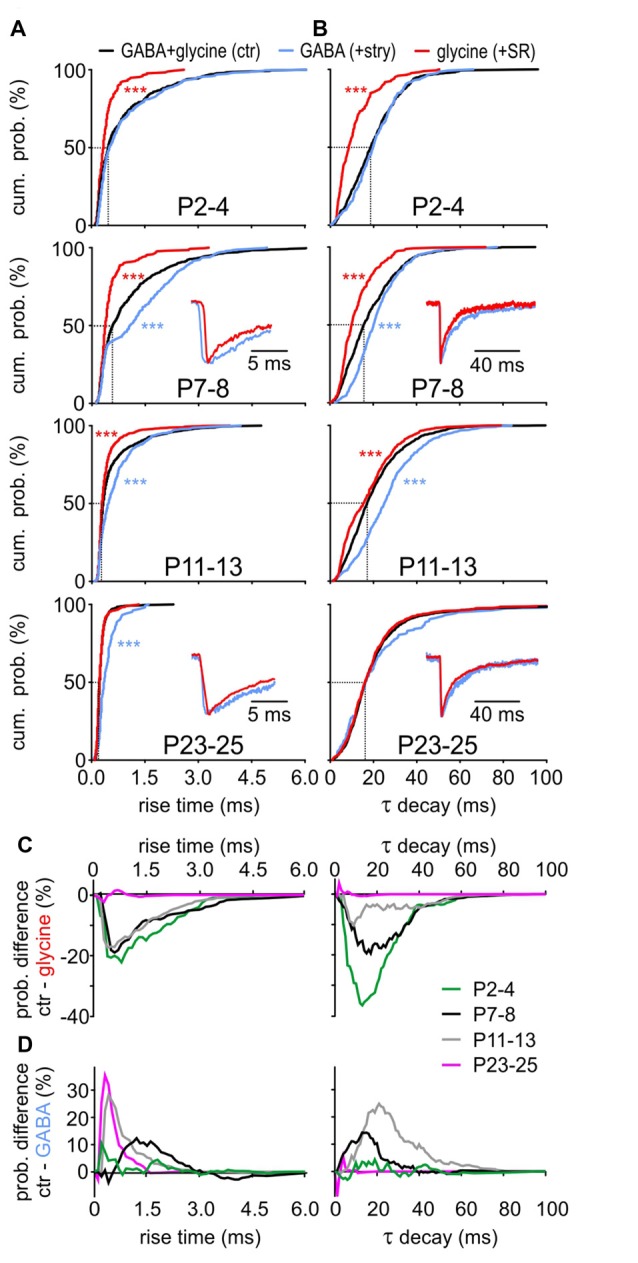
Contribution of GABA and glycine to mIPSC kinetics. **(A,B)** Mean CDFs of **(A)** rise times and **(B)** decay time constants at different postnatal ages for control, isolated GABAergic and glycinergic mIPSCs (ctr vs. transmitter: ***p < 0.001, K-S test). Insets show overlay of normalized median traces for representative cells at different ages. **(C,D)** Difference curves obtained by subtraction of glycinergic CDF **(C)** or GABAergic CDF **(D)** from respective control CDF for mIPSC rise time (left) and τ decay (right). The probability difference for rise time and τ decay between glycinergic and control mIPSCs decreased with maturation. Due to developmental shortening of the rise time for control events, the probability difference was progressively larger for maturing isolated GABAergic events (**D**, left). The decay time of glycinergic mIPSCs shortens with development **(B)** to reach values comparable to control at P23–25 (**D**, right).

Considering the developmental shift from GABA-dominating to glycine-dominating mIPSCs, we next investigated how GABA and glycine impact the kinetics of mIPSCs (Figure 4). During postnatal maturation, the rise time of GABAergic events was consistently slower compared to glycinergic events (Figure 4A, GABA vs. glycine: P2–4, P7–8, P11–13: *p* < 0.001, P23–25: *p* = 0.012, K-S test). GABAergic CDFs progressively shifted to shorter rise times to become as fast as solely glycinergic events at P23–25. Before (P7–8) and around hearing onset (P11–13), the control CDF was between the fast-rising glycinergic and slow-rising GABAergic events, indicating an equal contribution of both transmitters during this transient period. Similar to the difference in rise time, GABAergic events generally have slower decay time constants than the glycinergic events up to P11–13 (Figure 4B, GABA vs. glycine: P2–4, P7–8, P11–13: *p* < 0.001, K-S test). The decay time of control events was comparable to the values of GABAergic mIPSCs at P2–4, whereas both GABA_A_ and GlyRs seem to contribute to τ decay at P7–8 and P11–13. Notably, τ decay at P23–25 was similar for control, GABAergic and glycinergic mIPSCs (GABA vs. glycine: *p* = 0.16, GABA vs. ctr: *p* = 0.26, glycine vs. ctr: *p* = 0.81, K-S test). Combined, these results demonstrate distinct kinetics of isolated GABAergic and glycinergic mIPSCs which at different stages of development have different shares in controlling the time-course properties of miniature events.

At the central inhibitory synapses utilizing corelease of GABA and glycine, the decay time can have a bi-exponential profile with a fast (glycine) and a slow (GABA) component (Jonas et al., 1998; Chéry and de Koninck, 1999; O’Brien and Berger, 1999; Dumoulin et al., 2001; Gao et al., 2001; Russier et al., 2002; Nabekura et al., 2004; Awatramani et al., 2005). In the present study, however, bi-exponential decays were found in all three categories, i.e., control mIPSCs, isolated GABAergic, isolated glycinergic events. Pharmacological blockade did not eliminate either of the two exponential decay components. Therefore, the relative contribution of each transmitter to the control kinetics probability was assessed by subtraction of the isolated glycinergic CDF (+SR) or the isolated GABAergic CDF (+stry) from control CDF (Figures 4C,D). The difference curves indicate that up to P11–13 glycinergic events have shorter rise times than controls (Figure 4C *left*). At P23–25 the rise time is dominated by GlyR, evidenced by no difference between control and glycinergic events. On the other hand, there is a clear developmental increase in CDF difference between control and GABAergic events (Figure 4D *left*).

For control mIPSCs, τ decay is gradually decreasing during maturation (Figure 4B). In the same time span, control-glycinergic difference becomes smaller so that the events from both groups are similar at P23–25 (Figure 4C right). Conspicuously longer τ decay of GABAergic events causes the prominent difference from control at P7–8 and P11–13 (Figure 4D *right*). Considering the glycine content in 83.7 ± 11.0% of vesicles at P23–25, it is probably the major transmitter shaping the mIPSC decay at this age. This hypothesis is further supported by the low frequency of GABA-containing vesicles (Figure 2E) and by the selective GABA contribution to mIPSCs of large amplitudes evoked through corelease with glycine (Figures 3C,D).

### Development of GABA- and Glycinergic mIPSC Properties

**Figure 5:**
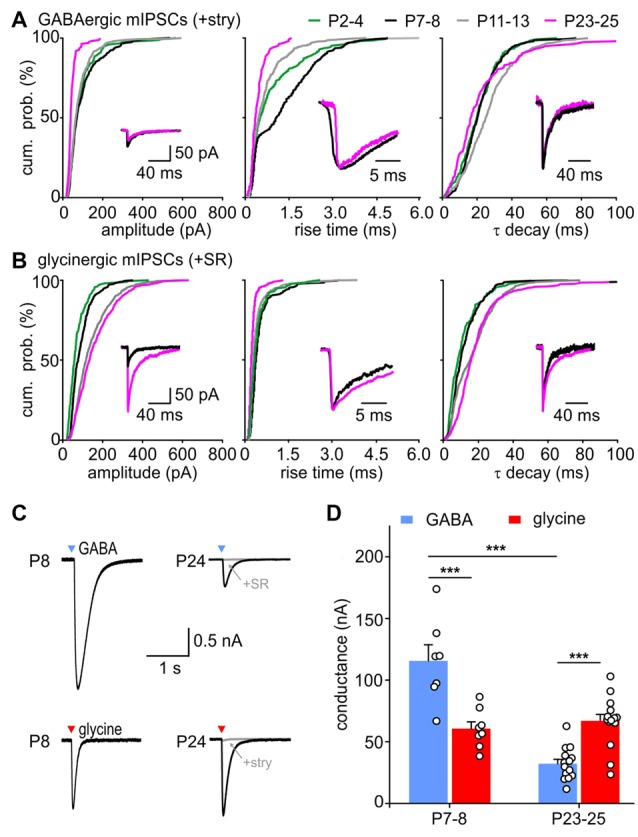
Maturation of GABA- and glycinergic mIPSC properties. **(A,B)** Developmental change of mean CDF for GABAergic **(A)** and glycinergic **(B)** mIPSC amplitudes (left), 10%–90% rise times (middle) and τ decay (right). Insets show median mIPSCs from representative SBCs at P7 (black) and P24 (magenta). The traces in the middle and right panel were normalized to peak to highlight the differences in mIPSC kinetics. **(C)** Whole cell currents evoked by a 5 ms pressure application of 0.5 mM glycine or GABA (*black*) at P8 and P24. Bath application of 0.5 μM strychnine or 20 μM SR95531 blocked the responses by 95% and 99%, respectively (*gray*). Arrowhead marks the time point of agonist application. **(D)** Population data of the conductance evoked by saturating concentrations of GABA and glycine at P7–8 and P23–25 shows a developmental decrease of GABA_A_ receptor (GABA_A_R) mediated effect by ~73% (***p < 0.001), whereas the glycinergic response did not change with age (*p* = 0.77, ANOVA). At P7–8 GABA evoked ~twofold larger responses than glycine (***p < 0.001, ANOVA, mean GABA/glycine ratio = 1.99 ± 0.26). At P23–25, glycine application evoked stronger conductance than GABA (***p < 0.001, ANOVA, mean GABA/glycine ratio = 0.50 ± 0.07).

The present data show a developmentally regulated contribution of GABA and glycine to the content of inhibitory vesicles. During the same time, however, also the properties of isolated GABAergic and glycinergic events change. This can be seen from changes of GABAergic and glycinergic mIPSCs, like the cumulative probabilities of their amplitudes, rise times and τ decay (Figure 5).

With maturation, isolated GABAergic mIPSC of amplitudes >100 pA gradually vanished (Figure 5A, P7–8 vs. P11–13: *p* < 0.05, P11–13 vs. P23–25: *p* < 0.01, K-S test). The events still found at P23–25 were mostly small in amplitudes (44.6 ± 3.3 pA, *n* = 6). In contrary, amplitudes of glycinergic events increased during development, so that amplitudes ranged from 25 pA to 600 pA in P11–13 and P23–25 cells (Figure 5B, P2–4 = 57.3 ± 10.2, *n* = 7; P7–8 = 77.2 ± 10.3 pA, *n* = 7; P11–13 = 122.9 ± 13.7, *n* = 8; P23–25 = 140.6 ± 14.4 pA, *n* = 6; effect of age: *p* < 0.001, pairwise comparisons: P2–4 vs. P11–13: *p* < 0.01, P7–8 vs. P23–25: *p* < 0.05, ANOVA; all CDF comparisons: *p* < 0.01, K-S test). The mIPSC kinetics, i.e., rise time and τ decay, were differently affected by age. Both GABA- and glycine-mediated events had longer rise times at P7–8 than at P2–4 (Figure 5A *middle*, GABA: P2–4 vs. P7–8 *p* < 0.001; Figure 5B *middle*, glycine: P2–4 vs. P7–8 *p* < 0.01, K-S test), but then again shortened up to P23–25 (GABA and glycine CDFs: P7–8 vs. P11–13 vs. P23–25: *p* < 0.001, K-S test; mean rise time of GABAergic in ms: P2–4 = 0.74 ± 0.15, P7–8 = 1.22 ± 0.32, P11–13 = 0.57 ± 0.18, P23–25 = 0.38 ± 0.05, effect of age *p* = 0.02, P7–8 vs. P23–25: *p* < 0.05, ANOVA; mean rise time of glycinergic in ms: P2–4 = 0.35 ± 0.03, P7–8 = 0.39 ± 0.0.05, P11–13 = 0.30 ± 0.03, P23–25 = 0.23 ± 0.02, effect of age *p* = 0.02, P7–8 vs. P23–25: *p* < 0.05, ANOVA). The decay time constants of GABAergic events showed a similar developmental trend, being the shortest at P23–25 (Figure 5A *right*; CDF comparisons P7–8 vs. P11–13 *p* < 0.001, P11–13 vs. P23–25: *p* < 0.001, K-S test). Shortening of τ decay could be due to the smaller amount of GABA released with maturity. This notion is supported by a positive correlation between the GABAergic mIPSC amplitude and the decay time constant in half of the tested cells (*r*_s_ = 0.38 ± 0.24, *n* = 6, *p* < 0.05 for 3/6 cells). For glycinergic events, the 10%–90% rise time decreased with maturity and showed no correlation with the mIPSC amplitude (*r*_s_ = −0.04 ± 0.13, *n* = 7, *p* > 0.05 for 6/7 cells). The lack of correlation rules out the possibility that the larger glycinergic mIPSCs could be prolonged by series resistance errors. Notably, the respective τ decay showed a conspicuous prolongation during development (Figure 5B, right panel; all comparisons *p* < 0.05, K-S test). The large glycinergic mIPSCs generally exhibited longer decay time constants, indicated by a positive correlation between the amplitude and τ decay in all cells (*r*_s_ = 0.44 ± 0.10, *n* = 7, *p* < 0.001 for 7/7 cells).

Events with long τ decay, as observed for glycinergic mIPSCs at P23–25, might be caused by a shift of synaptic inputs to dendritic locations, and ensuing filtering of currents recorded at the soma. To test this, Spearman correlation was calculated for the rise time and decay time of GABAergic and glycinergic mIPSCs at P7–8 and P23–25. No correlation was found for any of the tested groups (rise time vs. decay time: GABAergic mIPSCs P7–8 *r*_s_ = −0.02, *p* > 0.34 in 5/6 cells; P23–25 *r*_s_ = −0.02, *p* > 0.14 in 7/7 cells; glycinergic mIPSCs P7–8 *r*_s_ = 0.07, *p* > 0.35 in 5/6 cells; P23–25 *r*_s_ = 0.16, *p* > 0.12 in 6/6 cells). Together, these results rule out the dendritic filtering as a possible cause of developmental prolongation of mIPSCs.

The developmental changes of GABAergic and glycinergic mIPSC amplitudes can be caused by an increase/decrease of the transmitter amount in the presynaptic vesicles (Nabekura et al., 2004) and/or by a change of the postsynaptic GABA_A_R and GlyR recruitment (Nusser et al., 1997; Awatramani et al., 2005; González-Forero and Alvarez, 2005). Therefore, GABA and GlyR expression were examined by testing the sensitivity of SBCs to saturating concentrations (0.5 mM) of respective agonists at P7–8 (time point with largest GABAergic mIPSC amplitudes) and P23–25 (time point with the largest glycinergic and smallest GABAergic mIPSC amplitudes). The conductance elicited by pressure ejection of GABA decreased by 73% with age (Figure 5D, P7–8 = 115.6 ± 13.0 nS, *n* = 7, P23–25 = 32.2 ± 3.5 ns, *n* = 14, *p* < 0.001, ANOVA). In contrary, the glycine evoked conductance was similar in younger and older animals suggesting a stable expression level of GlyRs between the second and fourth postnatal week (P7–8 = 60.6 ± 5.5 ns, *n* = 8, P23–25 = 67.6 ± 5.4 ns, *n* = 15, *p* = 0.77, ANOVA). Considering these results, the prolongation of large mIPSCs during development might be due to an increase in the amount of glycine release. In addition to a developmental increase in amplitudes, glycinergic mIPSCs showed progressively shorter rise times and longer decay times. During the same period, GABAergic events became deliberately weaker with faster rise and decay times.

Analysis of mIPSC frequency 2 s before glycine application and 2 s after the I_Gly_ returned to baseline showed no difference (P23–25 before = 1.8 ± 0.5 Hz, after = 1.5 ± 0.5 Hz, *n* = 10, *p* = 0.24, paired *t*-test). This result suggests the lack of presynaptic GlyR on inhibitory terminals which could potentially affect release.

### Presynaptic Markers of GABAergic and Glycinergic Transmission are Partially Colocalized

**Figure 6:**
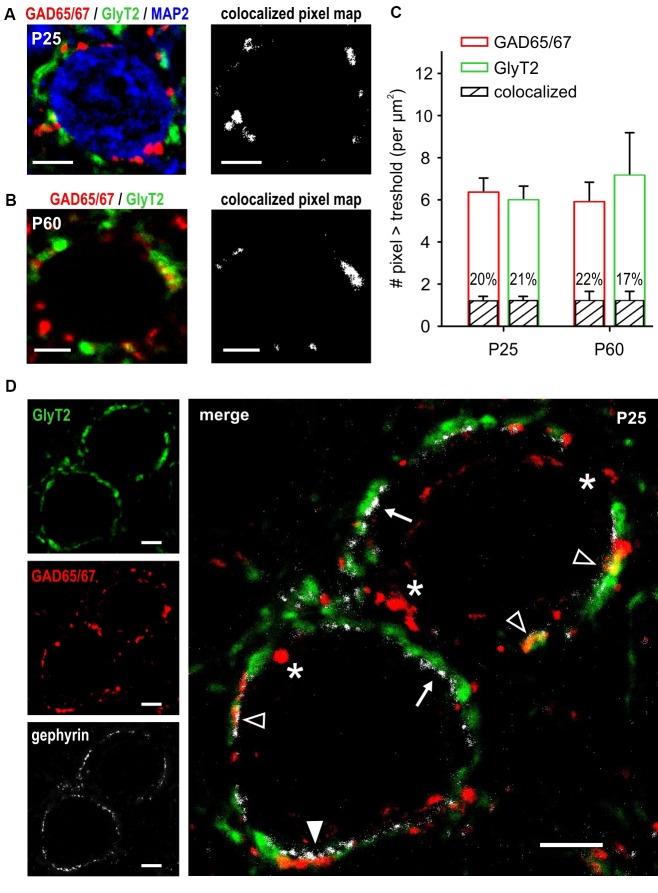
Partial colocalization of GABAergic and glycinergic presynaptic markers throughout adulthood. **(A,B)** Confocal images of GAD65/67 (red) and GlyT2 (green) labeling showing the allocation of putative GABAergic and glycinergic synaptic terminals at P25 **(A)** and P60 **(B)**. Additional staining with MAP-2 shows somatic localization of inhibitory terminals on the large SBCs (blue). Maps on the right show colocalized pixels (intensity above threshold in both channels) from the composite confocal images on the left. Partial colocalization of GAD65/67 and GlyT2 is present both at P25 and P60. **(C)** Population data showing GAD65/67 (red) and GlyT2 (green) pixels above respective threshold at P25 (*n* = 40 cells) and P60 (*n* = 24 cells). The amount of GAD65/67- and GLYT2-positive pixels is comparable within and between the age groups (*p* > 0.2, ANOVA). Black bars indicate the mean ± SEM number of colocalized pixels in both age groups. Percentages showing the relative fraction of colocalized pixels within the respective bar indicate a stable proportion of terminals releasing both GABA and glycine (P25 vs. P60, *p* = 0.8, ANOVA). **(D)** Gephyrin staining (white) indicates putative localizations of the postsynaptic GABA_A_R and/or GlyR. Merged image on the right shows gephyrin in apposition to GlyT2-positive terminals (arrows), to GAD65/67-positive terminals (white arrowhead), and to terminals colocalizing GlyT2 and GAD65/67 (empty arrowheads). Note the terminals expressing only GAD65/67 with no gephyrin staining on the postsynaptic site of the membrane (asterisks). Scale bars = 5 μm **(A–D)**.

The electrophysiological data revealed a developmentally regulated corelease of GABA and glycine that is also present at mature-like synapses. GAD65/67 and GlyT2 are reliable markers of GABAergic and glycinergic synapses (Fukuda et al., 1997; Poyatos et al., 1997), and both were previously found in inhibitory synapses co-releasing GABA and glycine (Tanaka and Ezure, 2004). Here, labeling was conducted at the stage when the development of inhibitory synapses is assumed to be complete (P25) and compared to a more advanced stage of maturity (P60). Additional MAP-2 staining allowed for morphological characterization of SBCs considering large cell soma and one or two solid primary dendrites (Figure 6A, left). In both age groups GAD65/67 and GlyT2 immunoreactivity was in apposition to the SBC cell soma, revealing conspicuous somatic GABAergic and glycinergic inputs (Figures 6A,B, left). Irrespective of age, majority of pixels were either GAD65/67- or GlyT2-positive. Still, some GAD65/67 positive pixels were also detected in the GlyT2 channel, indicating colocalization of both presynaptic markers (Figures 6A,B, right). Figure 6C shows the population analysis of pixels indicating GAD65/67, GlyT2 and colocalization of both around SBC somata at P25 (*n* = 40 cells) and P60 (*n* = 24 cells). At P25, comparable average numbers of GAD65/67 and GlyT2 pixels indicate matched numbers of GABAergic and glycinergic terminals (Figure 6C, pixel > tresh per μm^2^ P25: GAD65/67 = 6.4 ± 0.6, GlyT2 = 6.0 ± 0.6, *p* = 0.98, two way ANOVA). In both detection channels, 1.2 ± 0.2 pixels per μm^2^ were also detected in the respective other channel yielding about 20% colocalization (volume colocalized P25: GAD65/67 channel = 20.2 ± 1.5%, GlyT2 channel = 20.7 ± 1.6%). The total numbers of labeled pixels in either one of the detection channels were similar at both ages (pixel above pixel > tresh per μm^2^ P60: GAD65/67 = 5.9 ± 0.9, GlyT2 = 7.1 ± 1.9, GAD vs. GlyT2: *p* = 0.2; P25 vs. P60: GAD65/67 *p* = 0.82, GlyT2 *p* = 0.208, two way ANOVA). Also the number of pixels indicating colocalization did not change with promoted maturity (colocalized pixel per μm^2^ P60: 1.2 ± 0.5, P25 vs. P60: *p* = 0.8, two way ANOVA; volume colocalized P60: GAD65/67 channel = 22.4 ± 2.4%, GlyT2 channel = 17.5 ± 2.3%). In conclusion, these results suggest that in 1/5 of the labeled terminal area GABA is coreleased with glycine and that terminals engaging both GABA and glycine are kept at constant levels throughout early adulthood.

At P23–25, no mIPSCs were evoked by GABA only, although there were sole GAD65/67-positive puncta not colocalizing with GlyT2. This suggests the existence of GABAergic terminals lacking postsynaptic GABA_A_R-expressing sites. This notion was validated by gephyrin labeling of SBC, a protein contributing to the clustering of postsynaptic GABA_A_R and GlyR (Kneussel and Betz, 2000; Tyagarajan and Fritschy, 2014). Figure 6D shows abundant gephyrin expression in apposition to glycinergic (GlyT2 positive) and the terminals colocalizing GABA and glycine (GAD65/67 and GlyT2 positive). Notably, gephyrin staining was mostly absent at the postsynaptic site of the pure GABAergic terminals supporting the notion that at mature synapses some GABAergic terminals may not be involved in mediating a direct postsynaptic response via GABA_A_R.

## Discussion

This study shows a developmentally regulated shift of the neurotransmitter contained in inhibitory synaptic vesicles. Slice recordings taken from P1–P25 SBCs under superfusion of TTX and GABA_A_R- or GlyR-blocker revealed that the maturation profile of mIPSCs is determined by the developmentally regulated changes in GABA and glycine release. Early after birth, mIPSC properties are characterized by GABA signaling eliciting slow-rising and -decaying events of relatively small amplitudes. Around hearing onset and thereafter the inhibitory quanta predominantly contain glycine that triggers progressively larger and longer mIPSC with maturity. In addition, GABA corelease with glycine occurring consistently across all ages albeit with a low probability, evokes mIPSCs of particularly large amplitudes. Together, these results suggest that GABA as a primary transmitter released from immature terminals may initially play a developmental role, while it serves a modulatory function in maturity by increasing the inhibitory potency.

### GABA is the Initial Inhibitory Transmitter

In few SBCs, mIPSCs occurring at low-frequencies were detected at P1. This points to the early development of initial synaptic contacts, in accordance with anatomical studies showing fibers containing both GABA and glycine throughout the AVCN right at birth (gerbil: Gleich and Vater, 1998; rat: Luján et al., 2008). Early thereafter (P3–5), labeled inhibitory axons were shown to increasingly contact SBC somata. Up to the end of the first postnatal week, the percentage of cells in which mIPSCs could be recorded, and the corresponding mIPSC frequency, notably increased (Figure 1). Then, all inhibitory mIPSCs were mediated mostly by GABA with an additional glycinergic component in 34 ± 5% of the mIPSCS at P2–4 and 54 ± 12% of the mIPSCs at P7–8. In support of this notion, it was shown that GABAergic innervation of SBC somata develops prior to putative glycinergic terminals which have a weaker and delayed appearance in the cochlear nucleus of P1–P6 gerbils (Gleich and Vater, 1998).

Transient and developmentally-regulated GABAergic transmission was also described in the SOC (Kotak et al., 1998; Smith et al., 2000; Nabekura et al., 2004; Awatramani et al., 2005) and in spinal cord circuitries (Gao and Ziskind-Conhaim, 1995; Gao et al., 2001; Baccei and Fitzgerald, 2004). This raises the question about a potential developmental advantage of GABA over glycine action at immature synapses. During the first postnatal week, the outwardly directed Cl^−^ gradient, established by the activity of Na^+^-K^+^-2Cl^−^ cotransporter NKCC1, promotes a depolarizing GABA effect in SBCs (Milenković et al., 2007; Witte et al., 2014). This excitatory activity can trigger calcium signals through L- and R-type Ca^2+^ channels (Witte et al., 2014). Along the same lines, at mixed GABA/glycinergic synapses in the LSO, GABA was shown to have a higher potency in evoking intracellular Ca^2+^ signals (Kullmann et al., 2002). The longer GABAergic currents are presumably more effective in activating voltage gated Ca^2+^ channels than the glycinergic ones (Jonas et al., 1998; Chéry and de Koninck, 1999; Nabekura et al., 2004). Neurite growth and migration, synapse formation and synaptic plasticity generally involve calcium-triggered intracellular pathways (Bootman et al., 2001; Ben-Ari et al., 2007; Rosenberg and Spitzer, 2011). Besides evoking calcium signals, GABA- and glycine-mediated depolarization of LSO neurons can be suprathreshold and thereby elicit APs during the critical period of synaptic refinement, strengthening and tonotopic map formation (Kullmann et al., 2002; Kandler, 2004; Kullmann and Kandler, 2008). Accordingly, the structural refinement of MNTB-LSO synapses is restricted to the period when GABA and glycine are depolarizing (Kim and Kandler, 2003). Here, the underlying mechanism includes GABA-evoked long-term depression which precedes synapse elimination (Kotak and Sanes, 2000; Chang et al., 2003) and potentiation through GABA spillover to presynaptic terminals which causes synaptic strengthening (Weisz et al., 2016).

In the cochlear nucleus, conspicuous GABAergic signaling during the depolarizing phase may have an immediate implication for the development of inhibitory synapses on SBCs. Glycine is the dominant inhibitory transmitter in maturity, overtaking its function only after the staggered development.

### Shift from GABA- to Glycine-Dominated Transmission

Anatomical studies in mature guinea pigs and rats have shown glycinergic, GABAergic and terminals colocalizing GABA and glycine on SBCs (Juiz et al., 1996; Mahendrasingam et al., 2000, 2004; Luján et al., 2008). Consistent with these results, the present data show putative glycinergic and GABAergic inputs on SBCs and, in addition, ~1/5 of pixels colocalizing markers for both transmitters at P25 and P60 (Figure 6). Recordings after hearing onset show that mIPSC are either mediated by vesicles containing glycine only, or both glycine and GABA. No pure GABAergic mIPSCs were found presently and the lack of pure GABAergic IPSCs in juvenile SBCs had been reported earlier (Nerlich et al., 2014b). These findings indicate that some GABAergic terminals may lack their postsynaptic counterparts expressing GABA_A_R. Currently available literature does not provide evidence for presynaptic GABA_A_R in the cochlear nucleus (Lim et al., 2000; Chanda and Xu-Friedman, 2010). Moreover, presynaptic GlyR have been shown neither on the endbulb nor on inhibitory terminals, but their possible expression cannot be fully excluded. In our hands, presynaptic GABA_A_R/GlyR on the endbulb of Held would be expected to increase or decrease glutamate release, depending on whether the Cl^−^ gradient is depolarizing, as shown at the calyx of Held (Turecek and Trussell, 2001, 2002; Price and Trussell, 2006) or hyperpolarizing. In our experiments, pharmacological isolation of mIPSCs with GluR and GABA_B_R blockers does not allow investigation of respective effects. If the presynaptic GABA_A_R/GlyR were expressed on inhibitory terminals, pharmacological inhibition would cause a change in mIPSC frequency. However, the mIPSCs frequency remained unchanged at P2–4 and P7–8 under strychnine, suggesting no presynaptic GlyRs at these immature stages. On the other hand, SR95531 had no effect on spontaneous release at P23–25, indicating that GABA_A_R are probably not expressed at mature inhibitory terminals. In addition, mIPSCs frequency at P23–25 did not change after puff-applications of saturating glycine concentration, arguing against activation of presynaptic GlyR. Still, additional technically demanding experiments would be required to fully exclude the possibility that spillover of GABA/glycine is regulating release through presynaptic GABA_A_R/GlyR. Nevertheless, such effects would be unlikely to affect our conclusions about the developmental changes of vesicular content.

Both frequency and amplitude of mIPSCs evoked by vesicles containing glycine gradually increased between P2–4 and P23–25. This could be explained by a prolonged synaptogenesis of glycinergic terminals on SBCs and/or by an increase in the number of glycine-containing vesicles which has been shown to progress in a staggered fashion up to the fourth postnatal week (Luján et al., 2008). Generally, mIPSC amplitudes indicate the amount of GABA and glycine in presynaptic vesicles (Nabekura et al., 2004), but also the expression level of respective postsynaptic receptors (Lim et al., 1999; van Zundert et al., 2004; Awatramani et al., 2005). The latter is unlikely to account for the observed changes in glycinergic transmission, because the responses to puff application of saturating glycine concentrations were similar for P7–8 and P23–25 (Figure 5). The developmental increase in quantal size presumably reflects the increase in the vesicular glycine content, similar to what had been reported for developing LSO neurons (Nabekura et al., 2004), rather than a recruitment of postsynaptic GlyR (Keller et al., 2001; Awatramani et al., 2005; González-Forero and Alvarez, 2005). This is also evidenced by a comparison of synaptically evoked IPSCs (50 pulses at 100 Hz) in the same age groups which revealed similar amplitudes of the first events, but significantly more failures during ongoing stimulation in P7–8 SBCs (Figures 1D–F). Together, these data suggest an ongoing development of the synaptic machinery for glycine-releasing terminals extending well into the period after the onset of acoustically evoked signal processing.

The frequency of GABA-containing quanta remained stable during development (Figure 2), while the amplitudes of isolated GABAergic mIPSC reduced strongly after hearing onset (Figure 5). A down-regulation of postsynaptic GABA_A_R could account for this change, because also the responses to saturating GABA-puffs were strongly reduced between P7–8 and P23–25. The minor GABAergic contribution to mIPSC in maturity is consistent with earlier studies showing predominantly glycine-mediated synaptically evoked IPSC (Lim et al., 2000; Xie and Manis, 2013, 2014; Nerlich et al., 2014a). Thus, the maturation of inhibitory transmission to SBCs is characterized by a decrease of postsynaptic sensitivity to GABA counterbalanced by an increase in glycine content of presynaptic vesicles.

GABA does not seem to play merely a developmental role in SBCs. GABA_A_R has been shown to increase inhibitory strength during ongoing synaptic activity when GlyR currents undergo depression, thus providing slow, tonic-like inhibition to SBCs (Nerlich et al., 2014b). In the mixed GABA/glycine terminals of cartwheel cells in the dorsal cochlear nucleus, the vesicular transmitter content depends on the availability of cytosolic transmitters (Apostolides and Trussell, 2013). Synaptic activity at the endbulb of Held is likely to provide more glutamate as a substrate for GABA synthesis in inhibitory terminals. This could potentially increase the vesicular GABA content coinciding with excitatory activity, as shown for mixed inhibitory interneurons in the spinal cord (Ishibashi et al., 2013). At input rates of 100 Hz, GlyR but not GABA_A_R on mature SBCs were shown to saturate and/or desensitize, thereby significantly contributing to IPSCs depression (Nerlich et al., 2014a). During longer periods of acoustic stimulation, the GABAergic component could be particularly important to maintain the inhibitory effect. Present data indicate that the developmental down-regulation of postsynaptic GABA_A_R may be a factor limiting GABAergic strength in mature SBCs. This raises the question whether GABA may play some other role in addition to postsynaptic inhibition. Presynaptic GABA_B_R on the endbulb of Held terminals were shown to decrease glutamate release, thereby increasing the requirement for coincident activation of several presynaptic inputs to elicit postsynaptic AP firing (Chanda and Xu-Friedman, 2010). Similarly, GABA_B_R on inhibitory terminals were proposed to negatively regulate glycine release shortly after hearing onset (Lim et al., 2000). Therefore, using both GABA and glycine at mature inhibitory synapses on SBCs may be a mechanism to secure postsynaptic inhibition at different activity levels and to regulate transmitter release through a negative feedback mechanism.

### Slow Kinetics of mIPSCs throughout the Development

Synaptically evoked IPSCs in SBCs of gerbils and mice are remarkably slow (Xie and Manis, 2013; Nerlich et al., 2014b) due to activity-dependent delayed release, slow transmitter clearance, as well as glycine pooling and spillover (Nerlich et al., 2014a). These mechanisms jointly contribute to activity-dependent tonic-like inhibition (Xie and Manis, 2013; Nerlich et al., 2014a,b), also shown to be effective in granular cells of the dorsal cochlear nucleus (Balakrishnan et al., 2009). The presently measured τ decay of 17.2 ms for mIPSC in P23–25 SBCs is consistent with earlier studies (τ decay for single shock eIPSC ~24 ms Nerlich et al., 2014a,b) and suggests glycine rebinding or activation of remote receptors as mechanisms that may contribute to slow kinetics. This is supported by the data demonstrating that both the amplitude and the decay time constant of glycinergic events grow during development (Figure 5).

The expression of specific GlyR subtypes could also account for the slow mIPSCs in cochlear nucleus SBCs compared to stellate cells. These two types of principal neurons in the AVCN receive inhibitory inputs from the same sources, but employ inhibitory mechanisms operating on different time scales related to their different functions in sound processing (Xie and Manis, 2013). Differences in the kinetics and amplitude of inhibitory conductance can be attributed to distinct compositions of alpha1 and alpha2 subunits assembling GlyR (Wässle et al., 2009). Alpha1 is generally constituting the GlyR in maturity (Sato et al., 1995, 2000; Friauf et al., 1997), whereas alpha2 subunit is abundant in developing neurons of the brainstem and spinal cord (Akagi et al., 1991; Malosio et al., 1991; Sato et al., 1995; Friauf et al., 1997). Both subunits are only weakly expressed in the AVCN during the first postnatal week (Friauf et al., 1997; Piechotta et al., 2001), which is in agreement with presently shown postponed development of glycinergic inputs. Immunostaining in the cochlear nucleus indicated an overall increase in expression of the alpha1 subunit during first three postnatal weeks (Friauf et al., 1997). However, it is possible that alpha1 is largely assembling GlyR in stellate cells to provide large and fast conductance as observed in slice experiments (Xie and Manis, 2013). It is conceivable that heteromeric or homomeric GlyR on SBC include alpha2 subunits which would endow them with slow kinetics comparable to other alpha2 containing neurons, such as brainstem motoneurons in neonates (Singer et al., 1998), postnatal rat spinal cord neurons (Takahashi et al., 1992), and amacrine cells from mature retina (Veruki et al., 2007; Wässle et al., 2009).

In addition, dendritic filtering could potentially account for slow mIPSC kinetics in maturity (Rall, 1967; Rinzel and Rall, 1974). If this is the case, then a positive correlation between longer rise and decay times of synaptic events (Gardner et al., 1999; in our hands mIPSCs) should be expected. However, our data did not support this notion, since the two parameters were neither correlated at P7–8 nor P23–25. The developmental shortening of the rise time for mIPSCs is more likely mediated by a clustering of postsynaptic receptors on SBC somata (Lim et al., 1999), rather than a translocation of synapses from dendrites to the soma. Accordingly, a twofold longer rise time of eIPSCs vs. mIPSCs suggests asynchronous release during AP-driven synaptic activity (~0.44 ms vs. ~0.23 ms; Nerlich et al., 2014b and present study, respectively).

### Possible Sources of Inhibition

The onset of acoustically evoked inhibition in SBCs is delayed by few milliseconds with respect to excitation conveyed by the auditory nerve (Kuenzel et al., 2011; Keine and Rübsamen, 2015). This suggests a polysynaptic inhibitory pathway, possibly including D-stellate cells within the AVCN (Smith and Rhode, 1989; Campagnola and Manis, 2014) and/or tuberculoventral cells (TV) in the dorsal cochlear nucleus (Wickesberg and Oertel, 1990; Saint Marie et al., 1991; Campagnola and Manis, 2014), both of which receive a direct excitatory input from auditory nerve fibers. Both D-stellate and TV-cells seem well suited to provide broadly and symmetrically tuned inhibition with higher threshold and also slower onset and offset dynamics compared to the respective excitation (Kopp-Scheinpflug et al., 2002; Kuenzel et al., 2011; Nerlich et al., 2014b; Keine and Rübsamen, 2015; Keine et al., 2016). D-stellate cells convey broadly tuned glycinergic inhibition within the ipsilateral AVCN and send commissural projections to the contralateral AVCN (Cant and Gaston, 1982; Schofield and Cant, 1996; Doucet et al., 1999; Doucet and Ryugo, 2006; Campagnola and Manis, 2014). In slice experiments under pharmacological inhibition of GluR, single shock stimulation of the auditory nerve activates the tuberculoventral pathway and elicits an almost exclusively GlyR-mediated current in BCs (Wu and Oertel, 1986; Xie and Manis, 2013). These findings are consistent with the present data demonstrating predominantly glycinergic mIPSC after hearing onset.

Yet, electrical stimulation in the vicinity of SBC revealed also a GABAergic contribution to IPSCs (Lim et al., 2000) conspicuously emerging at higher stimulation rates (Chanda and Xu-Friedman, 2010; Nerlich et al., 2014b; Xie and Manis, 2014). The respective input may be lost in coronal or parasagittal slice preparations, due to disruption of the afferents from GABA and/or glycine containing neurons in the ventral and the lateral nucleus of the trapezoid body and in the dorsal pariolivary nucleus (Benson and Potashner, 1990; Schofield, 1991; Warr and Beck, 1996; Ostapoff et al., 1997). In guinea-pigs about 42% of periolivary neurons which project to the AVCN are GABA and glycine positive (Ostapoff et al., 1997). These tonotopically aligned descending projections from the SOC (Spangler et al., 1987) could be the potential source of the glycine- and GABA-containing quanta singled out in mIPSC recorded from SBCs. Along with the inhibitory circuit within the cochlear nucleus, they are well suited to provide an inhibitory feedback limiting the discharge rate of SBCs across a range of input levels throughout the neuron’s excitatory response area. Such inhibitory properties are likely to prevent level-dependent changes in response latencies which could interfere with the temporally precise binaural processing in the SOC (Keine and Rübsamen, 2015; Kuenzel et al., 2015).

## Author Contributions

JN, RR and IM contributed to the conception and design of experiments. JN performed experiments and analyzed the data. JN and IM wrote the manuscript and all authors approved the final version to be published.

## Conflict of Interest Statement

The authors declare that the research was conducted in the absence of any commercial or financial relationships that could be construed as a potential conflict of interest.
